# Supplementary material for: Seminoma and Embryonal Carcinoma Footprints Identified by Analysis of Integrated Genome-Wide Epigenetic and Expression Profiles of Germ Cell Cancer Cell Lines
Source: PLoS One. 2014 Jun 2;9(6):e98330. doi: 10.1371/journal.pone.0098330 (PMC4041891; doi:10.1371/journal.pone.0098330)
Supplement: File S1 — ZIP file containing DMRforPairs output for significant regions. Please start from the html files. (ZIP) [file pone.0098330.s008.zip › figures/1376.pdf]

RegionID: 1376, chr1:26233332−26233709−M_values

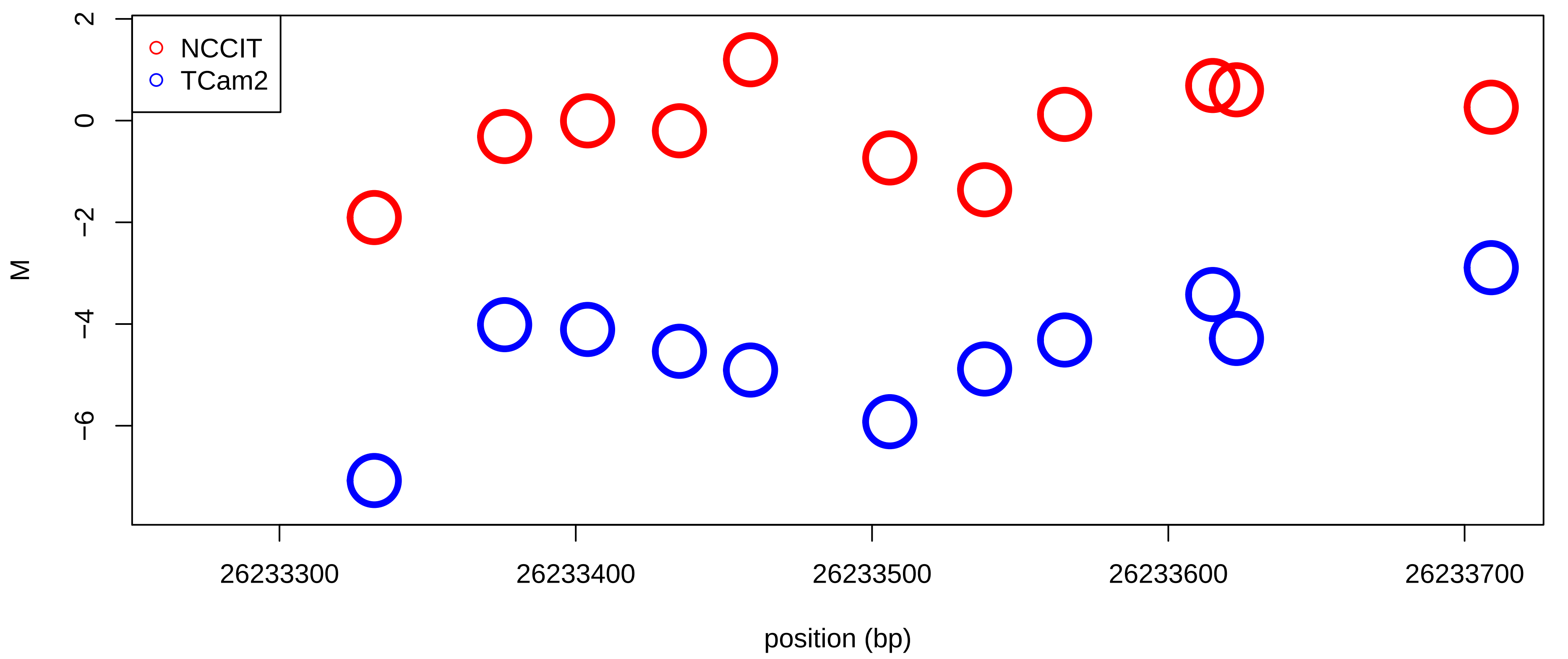

RegionID: 1376, chr1:26233332−26233709−Beta_values

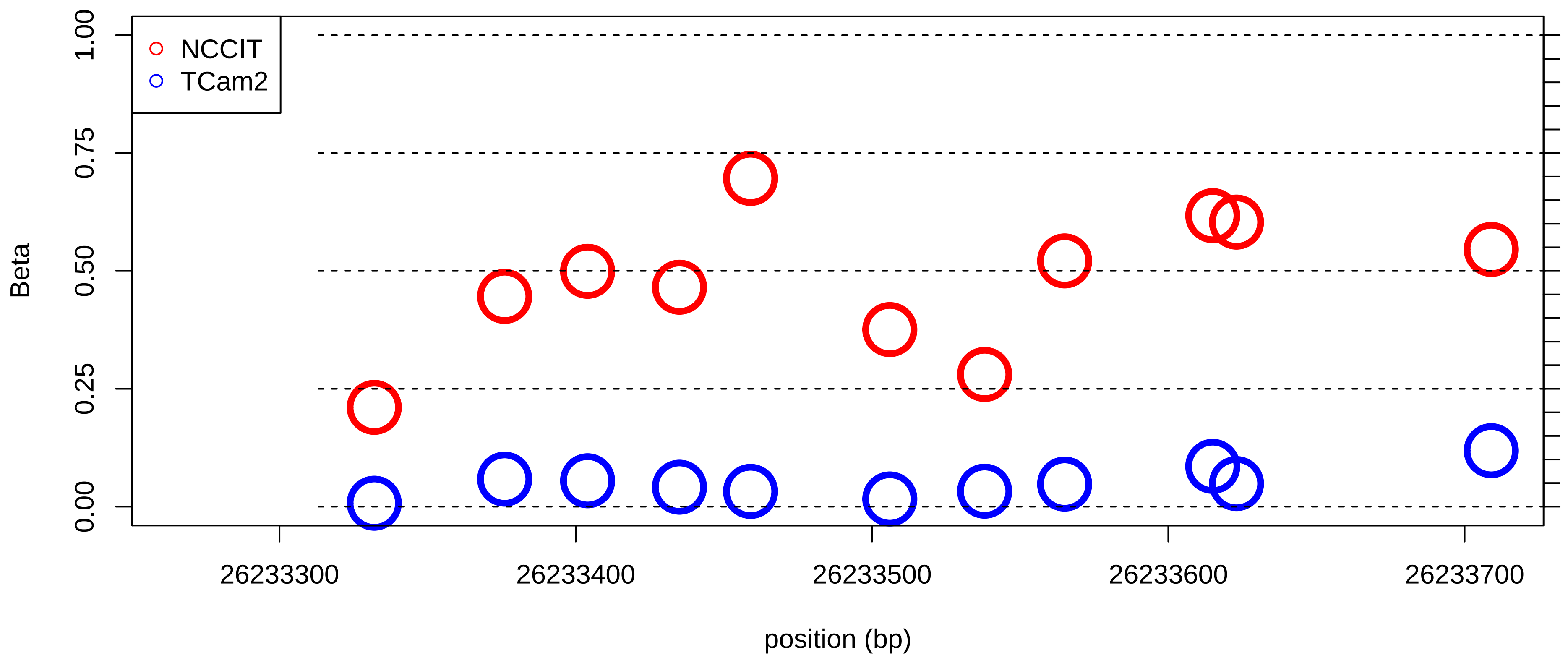

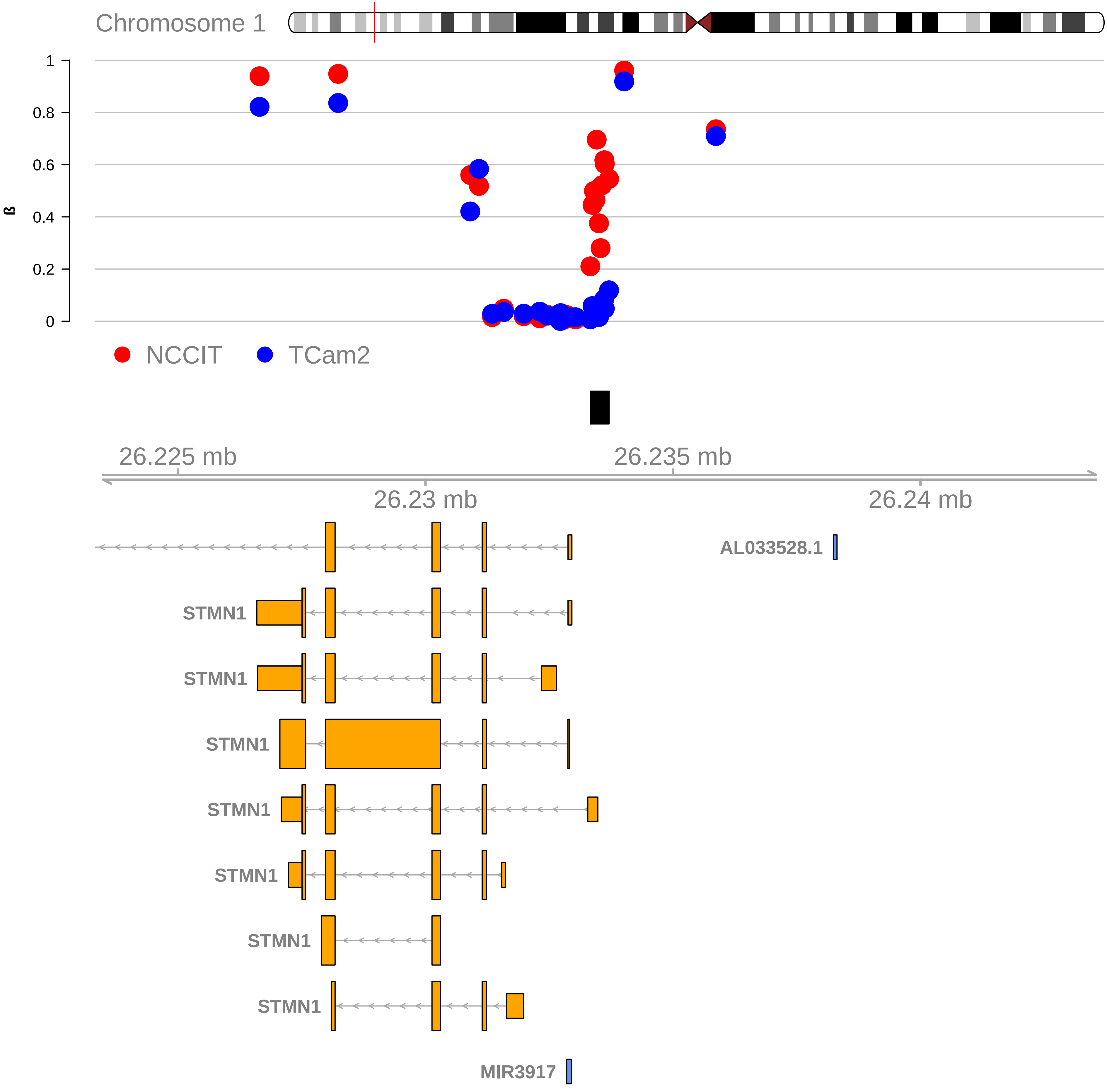

Chromosome 1
1
0.8
0.6
0.4
0.2
0
β
NCCIT
TCam2
26.225 mb
26.235 mb
26.23 mb
26.24 mb
AL033528.1
STMN1
STMN1
STMN1
STMN1
STMN1
STMN1
STMN1
MIR3917
